# *SNCA* Is a Functionally Low-Expressed Gene in Lung Adenocarcinoma

**DOI:** 10.3390/genes9010016

**Published:** 2018-01-04

**Authors:** Yuanliang Yan, Zhijie Xu, Xiaofang Hu, Long Qian, Zhi Li, Yangying Zhou, Shuang Dai, Shuangshuang Zeng, Zhicheng Gong

**Affiliations:** 1 Department of Pharmacy, Xiangya Hospital, Central South University, Changsha 410008, Hunan, China; yanyuanliang@csu.edu.cn (Y.Y.); xzj1322007@csu.edu.cn (Z.X.); huxiaofang006@sina.com (X.H.); qianlong@csu.edu.cn (L.Q.); dshuang@csu.edu.cn (S.D.); zss_93@163.com (S.Z.); 2 National Clinical Research Center for Geriatric Disorders (XIANGYA), Xiangya Hospital, Central South University, Changsha 410008, Hunan, China; 3 Department of Pathology, Xiangya Hospital, Central South University, Changsha 410008, Hunan, China; 4 Center for Molecular Medicine, Key Laboratory of Molecular Radiation Oncology of Hunan Province, Xiangya Hospital, Central South University, Changsha 410008, Hunan, China; peries@163.com; 5 Department of Medical Oncology, Xiangya Hospital, Central South University, Changsha 410008, Hunan, China; zhouyy423@163.com

**Keywords:** *SNCA*, lung adenocarcinoma, overall survival, therapeutic target

## Abstract

There is increasing evidence for the contribution of synuclein alpha (*SNCA*) to the etiology of neurological disorders, such as Parkinson’s disease (PD). However, little is known about the detailed role of *SNCA* in human cancers, especially lung cancers. Here, we evaluated the effects of *SNCA* on the occurrence and prognosis of lung adenocarcinoma (ADC). Comprehensive bioinformatics analyses of data obtained from the Oncomine platform, the human protein atlas (HPA) project and the cancer cell line encyclopedia (CCLE) demonstrated that *SNCA* expression was significantly reduced in both ADC tissues and cancer cells. The results of relevant clinical studies indicated that down-regulation of *SNCA* was statistically correlated with shorter overall survival time and post-progression survival time. Through analysis of datasets obtained from the Gene Expression Omnibus database, significant low levels of *SNCA* were identified in cisplatin-resistant ADC cells. Moreover, small interfering RNA (siRNA)-mediated knockdown of protein tyrosine kinase 7 (PTK7) elevated the expression of *SNCA* in the ADC cell lines H1299 and H2009. Our work demonstrates that low levels of *SNCA* are specifically found in ADC and that this gene may be a potential therapeutic target for this subset of lung cancers. Determination of the role of *SNCA* in ADC biology would give us some insightful information for further investigations.

## 1. Introduction

Lung cancer, the most frequent cause of cancer-related mortality worldwide, is responsible for approximately 1.6 million deaths annually. In most countries, adenocarcinoma (ADC) is the most frequent histological lung cancer subtype. Owing to late diagnosis, the 5-year overall survival rate for ADC is still very poor, ranging from 4% to 17% depending on the stage and regional differences [1,2]. Despite standard care and emerging targeted therapies, most ADC patients are often diagnosed at advanced stages and have a poor prognosis [3]. Thus, to improve clinical prognosis and outcomes, there is an urgent need for the discovery of novel molecular biomarkers for the early diagnosis of ADC.

Synuclein alpha (*SNCA*) was previously identified to be a pivotal modulator controlling the formation of misfolded protein aggregates (Lewy bodies) in Parkinson’s disease (PD) [4]. Recent studies have demonstrated that *SNCA* is also linked to various cancers, suggesting a probable role in cancer development. The transcription factor ΔNp63α can induce the expression of *SNCA*, thus promoting breast cancer invasion [5]. Hypermethylated *SNCA* in tumor tissue and stool samples might be useful as a biomarker for the noninvasive detection of colorectal cancer [6]. Through a microarray analysis of drug resistance-related microRNAs, Zou et al. found that down-regulation of *SNCA* is significantly correlated with multidrug resistance in ovarian cancer [7]. Strikingly, using a microarray meta-analysis approach, Karim et al. demonstrated that *SNCA* is one of the most significantly upregulated genes in prostate cancer patients treated with radiation therapy [8]. However, little is known regarding the effects of *SNCA* on the pathological processes of ADC and the mechanisms associated with its activity.

The aim of the present study was to assess the function and mechanism of *SNCA* in human lung ADC. Our data show that *SNCA* is significantly down-regulated in ADC tissues and cell lines. A Kaplan–Meier estimator identified *SNCA* as a putative prognostic factor for ADC. Mechanistically, we found a negative correlation between the expression of *SNCA* and epidermal growth factor receptor (EGFR). Further, we identified four possible EGFR phosphorylation sites in the SNCA protein sequence.

## 2. Materials and Methods 

### 2.1. Data Acquisition and Reanalysis Using Different Bioinformatics Methods

*SNCA* expression levels in ADC tissues and cell lines were examined using several bioinformatics web resources. These are summarized in Table 1.

Oncomine, a cancer microarray data-mining platform, is helpful for individual researchers to obtain gene expression signatures in human cancer tissues and cells [9]. UALCAN is an interactive and user-friendly web resource for the analysis of cancer transcriptomic data [10]. The Human Protein Atlas project enables users to create a map of protein expression patterns on a cellular level in multiple human tissues [11]. The CCLE project provides public access to a detailed genetic and pharmacologic characterization of approximately 1000 human cancer cells [12]. From these open bioinformatics platforms, *SNCA* expression profiles could be clearly identified in both human ADC tissues and cell lines.

Two algorithms, UALCAN and Kaplan-Meier Plotter, were used to evaluate the clinical relevance of *SNCA* in ADC by linking the cancer genome atlas (TCGA) clinical data to messenger RNA (mRNA) expression levels. In particular, UALCAN was used to analyze the association between *SNCA* levels and clinical characteristics of ADC patients. The Kaplan-Meier Plotter platform was used to quickly confirm disease prognosis, including overall survival (OS) time and post-progression survival time (PPS) [13].

Treatment-related transcriptome microarray datasets were downloaded from the gene expression omnibus (GEO) database under the accession numbers GSE50138 [14] and GSE21656 [15]. Normalized raw transcriptome data were subsequently reanalyzed to evaluate the influence of *SNCA* expression on the response of ADC patients to chemotherapy.

Using the default parameters, the Functional Enrichment analysis tool (FunRich) [16] was used to establish the interaction partners of *SNCA* as well as the related signaling pathways. The gene expression profiling interactive analysis (GEPIA) [17] and PhosphoNET databases [18] were used to study how SNCA may be regulated by EGFR in ADC.

### 2.2. Immnunohistochemistry Analysis of an Adenocarcinoma Tissue Array 

The ADC tissue array (LUC1601A) was purchased from Fanpu Biotech (Guangxi, China). Immnunohistochemistry analysis (IHC) was performed with the EnVision Detection Systems (Glostrup, Denmark) according to the manufacturer’s instructions. The antibody against *SNCA* (2647) was purchased from Cell Signaling (Danvers, MA, USA). Images of the sections were independently examined and differentially quantified by two pathologists. The staining intensity of each protein by IHC was scored as 0 (negative), 1 (weak brown), 2 (moderate brown), and 3 (strong brown). The extent of staining was scored as 0 (≤10%), 1 (11–25%), 2 (26–50%), 3 (51–75%), and 4 (>75%). The final staining score (0–3) was determined by the formula: intensity score × extent score.

### 2.3. Statistical Analyses

Proteins and mRNAs that were differentially expressed between cancerous and non-cancerous tissues or cells were analyzed using Student’s *t*-test with statistical package for the social sciences (SPSS) 12.0 software (IBM Analytics). Chi-square tests were used to analyze the associations between *SNCA* expression and clinicopathologic characteristics in lung cancer. Correlations between genes were assessed by Pearson correlation coefficient. *p* < 0.05 was considered statistically significant.

## 3. Results

### 3.1. Synuclein Alpha Is Down-Regulated in Adenocarcinoma Tissues and Cell Lines

**Figure 1:**
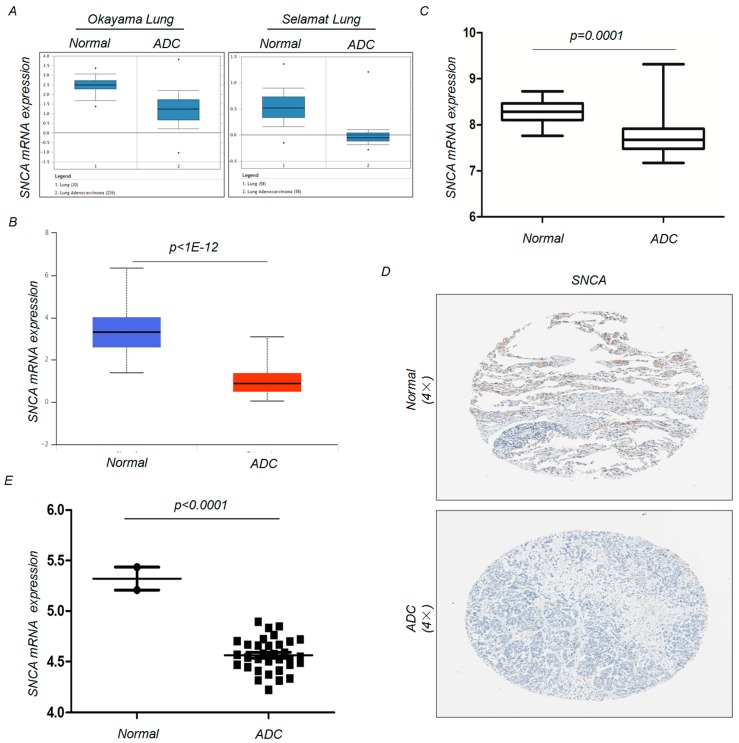
Analysis of *SNCA* expression levelsin ADC tissues and cell lines. (**A**) The messenger RNA (mRNA) expression of *SNCA* in Okayama Lung and Selamat Lung grouped by surrounding normal lung tissues and ADC; (**B**,**C**) The mRNA expression of *SNCA* was examined from the UALCAN and GEO public databases; (**D**) *SNCA* mRNA levels are significantly down-regulated in 33 LUAD cell lines, compared to 2 normal cell lines. Datawere obtained from the Cancer Cell Line Encyclopedia. (**E**) The Human Protein Atlas project shows representative immunohistochemical images from *SNCA* in ADC compared with noncancerous lung tissues.

**Figure 2:**
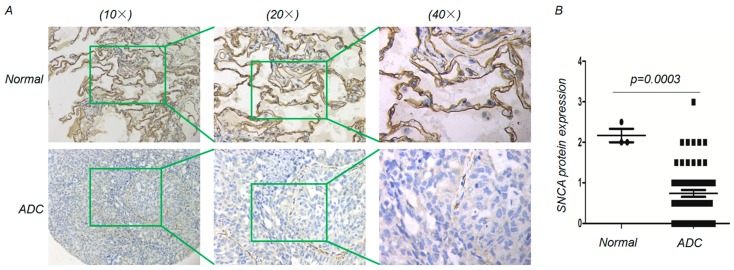
Downregulation of *SNCA* in ADC tissues array. (**A**) Immnunohistochemistry analysis (IHC) analysis was used to examine the level of *SNCA* expression in a commercial ADC tissue array. Representative images are shown; (**B**) The quartile graph indicate that the protein level of SNCA significantly downregulated in ADC patients.

To examine the changes in *SNCA* expression between ADC and adjacent non-tumor tissues, we analyzed *SNCA* expression profiles in five independent bioinformatics databases. First, we analyzed two microarray datasets from the Oncomine Platform, as shown in Figure 1A, and found that *SNCA* transcript levels were significantly reduced in tumor tissues. Similarly, using the UALCAN tool and a publicly available dataset downloaded from the GEO database (GSE10072) [19], we confirmed the down-regulation of *SNCA* transcripts in ADC tissues (*p* < 0.01) (Figure 1B,C). We next intended to verify this trend by clinicopathologic analyses. The IHC staining results from the Human Protein Atlas project suggested that *SNCA* is strongly or moderately expressed in normal lung specimens (positive rate of 50.00%) but weakly or negatively expressed in ADC tissues (positive rate of 8.33%) (Figure 1D). We next examined the expression of *SNCA* in ADC cancer cells. Through an exploration of the CCLE website, we discovered that, compared with immortalized lung epithelial cell lines, *SNCA* mRNA expression was significantly down-regulated in approximately 33 ADC cell lines (*p* < 0.0001) (Figure 1E). Additionally, we further verified the decreased trend using the IHC analysis on a commercial ADC tissue array, which contains 3 normal samples and 59 ADC samples (Figure 2A). Compared with the normal lung, SNCA protein level was low-expressed in ADC tissues (*p* = 0.0003) (Figure 2B). All these data provide evidence that decreased expression of *SNCA* contributes to ADC carcinogenesis, indicating its tumor suppressor function in lung cancers.

### 3.2. Synuclein Alpha Acts as a Putative Prognostic Factor in Adenocarcinoma

**Figure 3:**
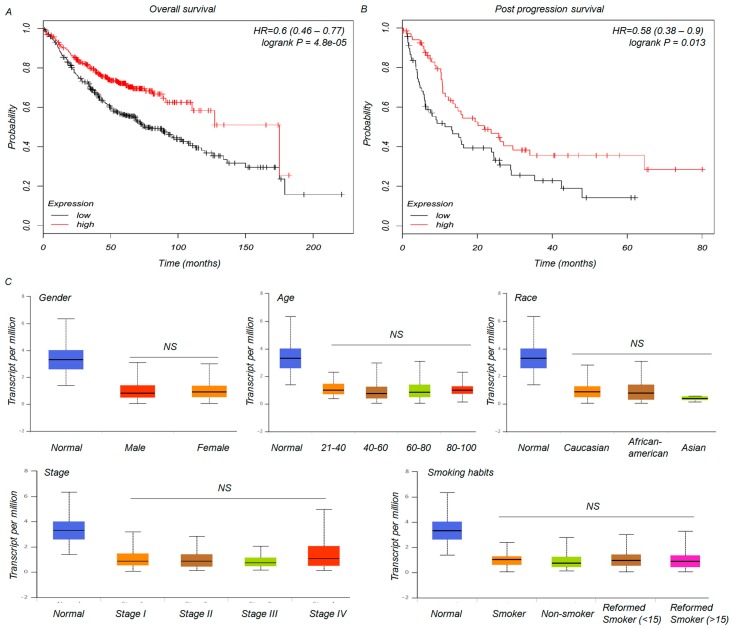
The association between *SNCA* expression and clinical characteristics of ADC patients. (**A**,**B**) Kaplan-Meier analysis of overall survival (OS) and post-progression survival time (PPS) in ADC patients based on *SNCA* expression; (**C**) The cancer genome atlas (TCGA) clinical data from the UALCAN web tool were used to categorize ADC patient characteristics according to their expression levels of *SNCA*, such as gender, age, race, etc.

To the best of our knowledge, despite the functional role of *SNCA* in human carcinogenesis [20], there have been no clear reports of the relationship between *SNCA* expression and the clinical prognosis of these diseases. We chose both OS and PPS as the monitoring indices for a clinical follow-up survey [21]. As shown in plots using the Kaplan-Meier Plotter platform, an intriguing TCGA analysis tool, down-regulation of *SNCA* expression was significantly correlated with shorter OS (*p* < 0.01) and PPS (*p* = 0.013) (Figure 3A,B). Then, we further studied the associations between *SNCA* levels and the clinical characteristics of ADC patients. Unexpectedly, based on the data from UALCAN, the expression of *SNCA* was not correlated with gender, age, race, stage, or smoking habits (Figure 3C). These results need to be further confirmed in a subset of tumor samples. Collectively, the findings mentioned above suggest that the reduced expression of *SNCA* in ADC patients might be a valuable prognostic factor.

### 3.3. The Role of Synuclein Alpha in Adenocarcinome Therapies

**Figure 4:**
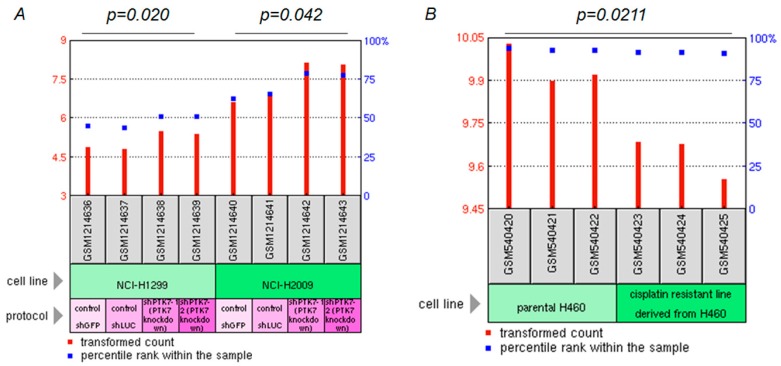
The influence of *SNCA* on the therapeutic responses of ADC patients. (**A**,**B**) Two treatment-related microarray datasets (GSE50138 and GSE21656) from the GEO database were used to evaluate the potential functions of *SNCA* expression on therapeutic effects in ADC patients.

Next, we used two treatment-related microarray datasets from the GEO database to further determine the potential role of *SNCA* in the therapeutic response of ADC patients. Previous studies identified *PTK7* as a survival gene in ADC and demonstrated that inhibition of *PTK7* by siRNA or monoclonal antibody could effectively impair tumor growth in vivo and in vitro [14,22]. From the GSE50138 dataset, we found that RNA interference-mediated attenuation of *PTK7* notably enhanced the expression of *SNCA* in two ADC cell lines, H1299 and H2009 (*p* = 0.020 and 0.042, respectively) (Figure 4A). Additionally, in the GSE21656 dataset, *SNCA* was significantly down-regulated in the cisplatin-resistant cell line (CDDP-R) as compared with the parental H460 cells (*p* = 0.0211) (Figure 4B). These results provide insight into the functional role of *SNCA* in lung neoplasia and show that changes in *SNCA* expression levels might be involved in cancer treatment.

### 3.4. Synuclein Alpha Is Associated with the Epidermal Growth Factor Receptor Signaling Pathway in Adenocarcinoma Cells

**Figure 5:**
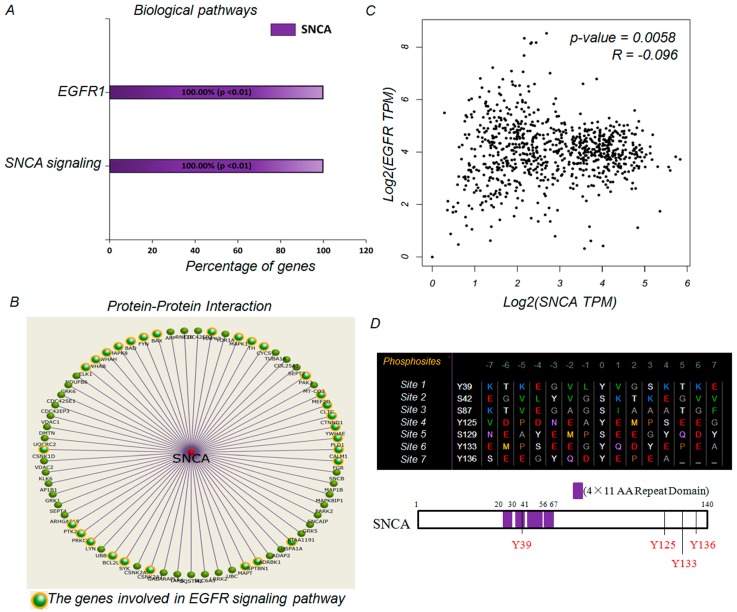
*SNCA* is modulated by the *EGFR* signaling pathway. (**A**) FunRichsoftware identified the EGFR1 signaling pathway as one of the top significant GO biological processes associated with *SNCA*; (**B**) The PPI network of *SNCA* interaction partners, created using the FunRich algorithm; (**C**) The GEPIA tool showed a negative correlation between *SNCA* and *EGFR* transcript levels in ADC tissues; (**D**) Putative EGFR phosphorylation sites were predicted in the SNCA protein sequence using the PhosphoNET website (http://www.phosphonet.ca/).

Previous studies have demonstrated that *EGFR*, a well-known oncogenic driver, contributes to the initiation and progression of lung cancer [23,24,25]. A subset of this investigation aimed to confirm whether there is a direct association between *SNCA* and the *EGFR* signaling pathway. Functional enrichment analysis performed using the FunRich software tool indicated that one of the main biological pathways associated with *SNCA* is the *EGFR1* signaling pathway (Figure 5A). Through analysis of the protein-protein interaction (PPI) network, we found that nearly half of all *SNCA* interaction partners were involved in modulating the *EGFR* signaling pathway, including mitogen-activated protein kinase (*MAPK*), phospholipase D (*PLD*), *PTK2*, etc. (Figure 5B). All these results demonstrate a potential association between *SNCA* and the EGFR signaling pathway. To further study the effects of *SNCA* and *EGFR* in ADC biological processes, we conducted a correlation analysis using GEPIA [17], a bioinformatics research platform for the profiling and interactive analysis of cancerous gene expression based on the TCGA and Genotype-Tissue Expression (GTEx) [26] databases. Our findings showed a significant negative trend of *SNCA* and *EGFR* transcript levels in ADC patient tissues (*p* = 0.0058, correlation coefficient = −0.096) (Figure 5C). To the best of our knowledge, EGFR could modulate its substrates (such as *SCD1*) through phosphorylation, thereby promoting lung cancer growth [27]. Next, we used a web server, PhosphoNET, to screen seven phospho-sites in the SNCA protein sequence. Among these putative sites, the residues Y39, Y125, Y133 and Y136 are the most likely targets for phosphorylation by EGFR (Figure 5D), suggesting that *SNCA* could act as a promising target of the oncogenic receptor tyrosine kinase *EGFR*. Taken together, all the findings mentioned above demonstrated a link between *SNCA* and the *EGFR* signaling pathway in ADC.

## 4. Discussion and Conclusions

This study was conducted to determine the feasibility of *SNCA* as a potential biomarker in ADC patients. Our study is the first to analyze the differential expression of *SNCA* in ADC tissues using different public datasets and examine its roles in cancer-related signaling pathways to identify its likely biological significance in carcinogenesis. Using the GEO and TCGA datasets, we have revealed that *SNCA* is significantly down-regulated in ADC tissues and that its expression levels are positively correlated with the OS and PPS of cancer patients.

*SNCA* is a pivotal modulator controlling the onset of PD and dementia with Lewy bodies [28,29,30]. Impairment of the autophagy-lysosomal pathway in the diseased brain has been shown to not only limit intracellular degradation of misfolded *SNCA* aggregates but also to lead to a detrimental microenvironmental response due to enhanced *SNCA* secretion, thus promoting PD pathology [31]. A neuroprotective modulator, 6-Bio, could clear the toxic *SNCA* aggregates by dramatically enhancing autolysosomal formation in yeast and mammalian cell lines [32]. Nilotinib, a brain-penetrant tyrosine kinase inhibitor, also facilitates the autophagic clearance of *SNCA* in transgenic and lentiviral gene transfer models, thus improving the major PD symptoms [33]. All of the findings mentioned above highlight the important role of the autophagy-lysosomal pathway in *SNCA* degradation, identifying exciting new options for PD therapy. However, recent studies have demonstrated the potential role of *SNCA* in the pathological processes underlying human cancer. B-cell lymphoma 2 (BCL2)-interacting killer (apoptosis inducing) (BIK) controls the expression of autophagy-associated transcripts, such as *SNCA*, that participate in tumor progression in the breast cancer cell line MDA-MB-231 [34]. *SNCA* methylation in the stool could also serve as a promising biomarker for the detection of colorectal cancer [6]. To date, there are no studies of the function of *SNCA* in association with lung cancer. In our study, using bioinformatics analyses of public datasets, we have illustrated the key tumor suppressive roles of *SNCA* in ADC samples. Furthermore, we have provided the first direct evidence of improved OS and PPS in ADC patients with high expression of *SNCA*. Further studies are needed to more comprehensively explore the detailed molecular mechanisms of this altered biomarker in the progression of ADC. 

Currently, biological pathway analyses have stated clearly that the EGFR1 and α-synuclein signaling pathways are the most significant pathways modulated by *SNCA*. Similarly, some previous reports also confirm the key roles of EGFR signaling in the etiology of human cancers, including lung cancer. EGFR can be phosphorylated on a regulatory tyrosine, constitutively activating the receptor in a ligand dependent or independent manner, thereby inducing tumor progression [35]. Loss of sortilin in the human lung cancer cell line A549 accelerates cell proliferation by sustaining EGFR signaling at the cell surface, ultimately promoting tumor growth [36]. Analysis of protein phosphorylation using the PhosphoNET algorithm allowed us to screen five probable EGFR phosphorylation sites in the full-length SNCA protein sequence. Meanwhile, a negative association between SNCA and EGFR expression could be clearly identified from ADC patient tissue data from the TCGA database. These results suggested that active EGFR might down-regulate the expression of SNCA by protein phosphorylation. Further research is needed to elucidate the detailed mechanisms of EGFR on the modulation of SNCA in ADC. 

In the present study, we identified *SNCA* as a promising molecular marker with prognostic value in ADC. Analysis of the public lung cancer data provides a meaningful method to screen key genes associated with the onset of human malignant diseases in the future. Moreover, the candidate differentially expressed gene, *SNCA*, could be a novel biomarker for the biological behavior of ADC, serving as a putative tumor suppressor gene.

## Figures and Tables

**Table 1:** The main bioinformatics tools used to analyze the functions of synuclein alpha (*SNCA*) in adenocarcinoma cells (ADC) biological processes.

Databases	Samples	URL	Refs
Oncomine	Tissues/Cells	https://www.oncomine.com/resource/login.html	[9]
UALCAN	Tissues	http://ualcan.path.uab.edu/index.html	[10]
Human protein atlas project	Tissues/Cells	http://www.proteinatlas.org/	[11]
CCLE	Cells	https://portals.broadinstitute.org/ccle_legacy/home	[12]
Kaplan-Meier plotter	Tissues	http://kmplot.com/analysis/	[13]
GEO	Tissues/Cells	https://www.ncbi.nlm.nih.gov/geoprofiles/	[14,15]
FunRich	-	http://www.funrich.org/	[16]
GEPIA	Tissues	http://gepia.cancer-pku.cn/	[17]

CCLE: Cancer cell line encyclopedia; GEO: Gene expression omnibus; GEPIA: Gene expression profiling interactive analysis.
